# Mesoporous Silica Modified with Polydopamine and Zinc Ions as a Potential Carrier in the Controlled Release of Mercaptopurine

**DOI:** 10.3390/ma16124358

**Published:** 2023-06-13

**Authors:** Mariusz Sandomierski, Martyna Chojnacka, Maria Długosz, Monika Pokora, Joanna Zwolińska, Łukasz Majchrzycki, Adam Voelkel

**Affiliations:** 1 Institute of Chemical Technology and Engineering, Poznan University of Technology, ul. Berdychowo 4, 60-965 Poznań, Poland; martyna.j.chojnacka@student.put.poznan.pl (M.C.); maria.dlugosz.pl@gmail.com (M.D.); adam.voelkel@put.poznan.pl (A.V.); 2 Center for Advanced Technologies, Adam Mickiewicz University, Poznań, ul. Uniwersytetu Poznańskiego 10, 61-614 Poznań, Poland; monika.pokora@amu.edu.pl (M.P.); joakol1@amu.edu.pl (J.Z.); lukmaj@amu.edu.pl (Ł.M.)

**Keywords:** acute lymphoblastic leukemia, 6-mercaptopurine, polydopamine, drug delivery, zinc

## Abstract

Mercaptopurine is one of the drugs used in the treatment of acute lymphoblastic leukemia. A problem with mercaptopurine therapy is its low bioavailability. This problem can be solved by preparing the carrier that releases the drug in lower doses but over a longer period of time. In this work, polydopamine-modified mesoporous silica with adsorbed zinc ions was used as a drug carrier. SEM images confirm the synthesis of spherical carrier particles. The particle size is close to 200 nm, allowing for its use in intravenous delivery. The zeta potential values for the drug carrier indicate that it is not prone to agglomeration. The effectiveness of drug sorption is indicated by a decrease in the zeta potential and new bands in the FT-IR spectra. The drug was released from the carrier for 15 h, so all of the drug can be released during circulation in the bloodstream. The release of the drug from the carrier was sustained, and no ‘burst release’ was observed. The material also released small amounts of zinc, which are important in the treatment of the disease because these ions can prevent some of the adverse effects of chemotherapy. The results obtained are promising and have great application potential.

## 1. Introduction

The most common pediatric malignancy is acute lymphoblastic leukemia (ALL) [1]. This disease is responsible for approximately 25% of all childhood cancers and 75–80% of childhood leukemias [2,3]. Survival and cure rates have improved over the past few decades due to the optimal use of antileukemic drugs [4,5]. One of the drugs used in this disease is 6-mercaptopurine (MERC) [6]. MERC is a drug with anti-inflammatory, immunosuppressive, and cytotoxic properties, and its action is dose-dependent. In high doses, it has immunosuppressive and cytotoxic properties, while in small doses, it acts as an anti-inflammatory drug [7,8]. This drug is also used in other diseases: ulcerative colitis and Crohn’s disease [9,10]. A problem with MERC therapy is its low bioavailability, ranging from 10% to 50%, with an average value of 16% [11]. The low bioavailability is due to the short plasma half-life of MERC, which is approximately 1 to 3 h [12]. This problem can be solved by preparing the drug delivery system that releases the drug in lower doses but over a longer period of time. Many modern drug delivery systems have been described in the literature in the last few years. An interesting example are polysialic acid-based drug delivery systems [13]. These systems have been used, for example, in the delivery of drugs in the treatment of cancer and rheumatic and neurological diseases. Particularly important drug delivery systems are those that release the drug in response to stimuli [14]. The stimuli that affect drug release can be divided into: internal stimuli (reactive oxygen species, enzyme, shear stress, and pH) and external stimuli (light, ultrasound, and magnetism). Stimuli-responsive drug delivery systems have been used, for example, in the treatment of atherosclerosis [14]. The use of drug delivery systems is particularly important for the stimulator of interferon genes. The stimulator of interferon genes shows promising clinical activity in infectious diseases and tumors [15]. However, the lack of targeting capability and intracellular stability of the stimulator of interferon genes agonists severely limits the therapeutic efficacy [15]. Recently, drug delivery systems (e.g., liposomes, polymeric nanoparticles) have overcome these delivery barriers.

Scientists have already tried to create drug delivery systems for MERC. Part of the proposed carriers are based on the formation of disulfide bonds between the carrier and the drug [16]. An example is the UiO-66-(SH)_2_ metal organic framework-based drug carrier prepared by Gong et al. [17]. In another publication, Talib et al. prepared biotinylated carbon dots nanoparticles as a drug delivery system [18]. The major disadvantage of the glutathione-sensitive drug delivery system is that the drug will only be released in the presence of a sufficient amount of glutathione. If the amount of glutathione is insufficient, the drug will be strongly bound to the carrier and will be removed from the body with it. The drug is also delivered using another metal organic framework. Mosavi et al. prepared NMOF-5 coated with chitosan and used it as a carrier [19]. In another work, Kaur et al. obtained a carrier based on a zeolitic imidazolate framework with the drug encapsulated inside the particle [20]. In both cases, the release is related to the dissolution of the metal organic framework.

An interesting alternative to this type of carrier may be carriers that release the drug under the influence of human body fluids. Due to the interaction with MERC, such a carrier should contain zinc in its structure [21]. The material that can most likely be used for this application is polydopamine-coated mesoporous silica [22,23]. Mesoporous silica is a drug carrier widely used by scientists around the world. Until now, mesoporous silica has been used for active substances such as fucoxanthin and curcumin [24,25,26,27]. The silica surfaces in these publications were modified, for example, with polyphenols, fucoidan, and alginates, which contributed to improving the properties of the carrier. Another type of modification of mesoporous silica is modification with polydopamine. Polydopamine is a biological polymer inspired by mussels [28]. It is used in biomedical applications due to its low cytotoxicity and exceptional biocompatibility [29]. Such carriers have already been used in the administration of avermectin, desipramine, minocycline, quercetin, and doxorubicin [30,31,32,33,34,35]. The use of carriers resulted in the prolonged release of drugs. To the knowledge of the authors of this work, this carrier has never been used as a carrier for MERC. This is surprising because this carrier has a very high potential due to the possibility of adsorbing zinc ions on its surface to which the drug can then attach [36]. Furthermore, the supply of zinc ions in the ALL treatment is very important because these ions can prevent some of the adverse effects of chemotherapy in children with leukemia, improving their quality of life [37]. Low blood levels of zinc are often noted in ALL and could be supplemented to some extent by the carrier [38].

The preparation of a carrier based on mesoporous silica coated with polydopamine, zinc ions, and the adsorbed drug may bring many benefits in the treatment of ALL. This material has not been previously described in the literature. Mesoporous silica is considered non-toxic and is used in drug delivery, so it is an ideal material on which polydopamine will be deposited [33,34]. Polydopamine, on the other hand, is a material modeled on natural organisms, and its biocompatibility with respect to the human body has been proven [39]. Zinc ions are considered toxic in some reports in the literature, but toxicity occurs only at high concentrations, whereas in low concentrations, is an essential trace element for humans [40,41]. These ions are increasingly used in biomaterials due to their properties that support processes in the human body [42,43]. Importantly, the effectiveness of these ions in therapy against ALL has been proven [37,38]. The last component of such a system is the active substance. Mercaptopurine is a drug that has been proven to work against ALL [2]. Combining the release of zinc and mercaptopurine in a controlled manner from a biocompatible carrier can achieve a double therapeutic effect. This type of material has not been described in the literature so far and may result in significant progress in the development of ALL treatment. The only material with a similar effect is the carrier in the form of zinc zeolite, shown previously by us, in which mercaptopurine was retained and released in a controlled manner [7].

**Figure 1:**
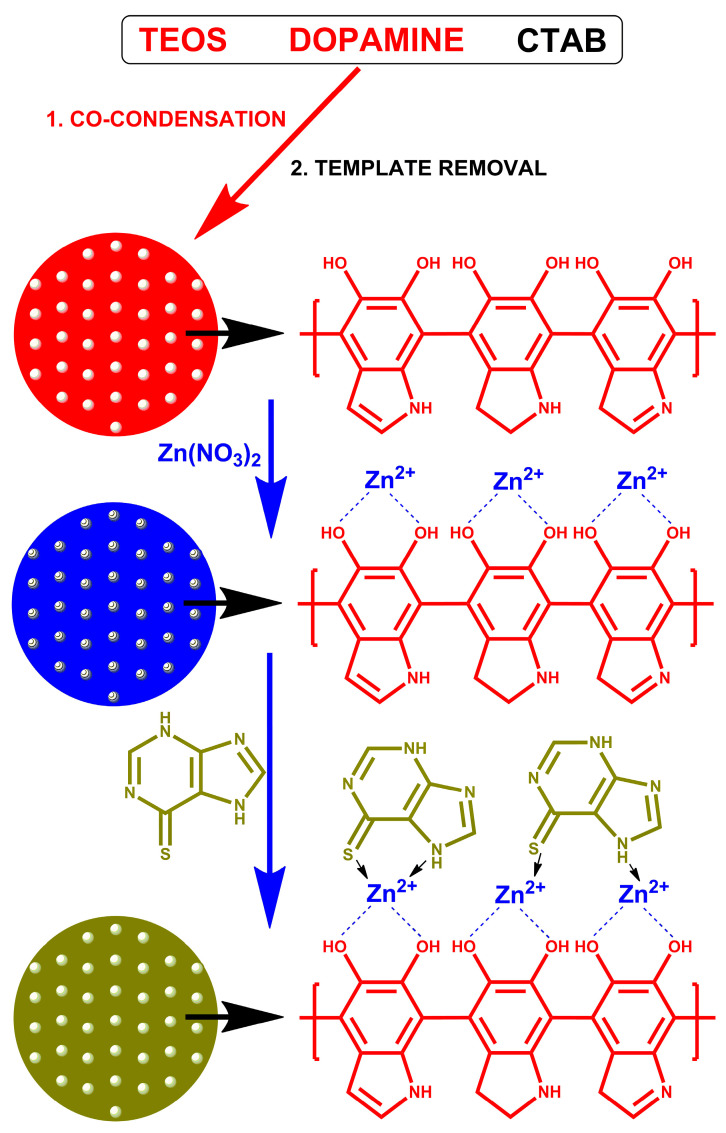
Schematic illustration of mSiO2-PDA-Zn-MERC synthesis.

In this work, a drug carrier for mercaptopurine was prepared on the basis of mesoporous silica coated with polydopamine. Zinc ions were adsorbed on the surface of the polydopamine-coated silica. The drug was retained on the carrier surface by the interaction with zinc. As a result of drug–carrier interactions, the release will be slow, which may increase the bioavailability of the drug. The material was characterized both before and after drug sorption. The study determined whether the drug was adsorbed and at what time it was released. The mechanism of drug adsorption is shown in Figure 1.

## 2. Materials and Methods

### 2.1. Chemical Compounds

Cetyltrimethylammonium bromide (CTAB, 99%), ammonia solution (25%), tetraethyl orthosilicate (TEOS, 98%), dopamine hydrochloride (DA), zinc nitrate hexahydrate (98%), 6-mercaptopurine (MERC), tris (hydroxymethyl) aminomethane (TRIS), (99.8%), sodium chloride (99%), sodium bicarbonate (99%), sodium sulfate (99%), potassium phosphate dibasic trihydrate (99%), and potassium chloride (99%) were purchased from Sigma-Aldrich (St. Louis, MO, USA). Hydrochloric acid (36–38%) was purchased from Avantor Performance Chemicals (Gliwice, Poland). The materials were used without further purification.

### 2.2. Preparation of Polydopamine-Modified Mesoporous Silica

The synthesis of polydopamine-modified mesoporous silica was carried out according to the methodology proposed by Shen et al. [30]. Co-condensation was used during the synthesis [30,44]. In the first step of synthesis, CTAB (0.9 g), ammonia solution (6.8 mL, wt = 25%), ethanol (60 mL), and deionized water (300 mL) were added to the flask. All ingredients were mixed with a mechanical agitator at a temperature of 70 °C for 30 min. Then, TEOS (2.84 g) was added dropwise to the solution. After stirring for 5 min, DA (65.0 mg) was added to the solution and left for 24 h. The resulting material was centrifuged (3000 rpm) for 5 min, washed with ethanol, and dried at 60 °C. The next step was to wash the material in an ethanolic solution of ammonium nitrate (10 mg/mL, 400 mL) at 60 °C for 8 h. After 8 h, the material was centrifuged. The washing step was repeated three times to remove impurities. Finally, the material was washed three times with pure ethanol, centrifuged, and dried at 60 °C for 24 h.

### 2.3. Zinc Adsorption on Polydopamine-Modified Mesoporous Silica

Zinc cations adsorption was carried out by mixing 30 mL of 0.1 M zinc nitrate solution with 1 g of polydopamine-modified mesoporous silica for 24 h. Then, the material was centrifuged (8000 rpm). The whole process was repeated three times. Subsequently, the material was washed three times with distilled water to remove excess zinc nitrate and then dried in an oven for 24 h at 60 °C.

The material after ion adsorption was named mSiO2-PDA-Zn.

### 2.4. Mercaptopurine Sorption

MERC sorption was initiated by introducing 15 mg of mSiO2-PDA-Zn into a 2 mL vial. Each vial was filled with 1 mL of MERC solution at a concentration of 0.015 mg/mL (the drug was dissolved in 0.1 M TRIS-HCl buffer at pH = 7.4). The samples were mixed on a laboratory rotator mixer (speed 50 rpm) for one week at room temperature. The carriers were then centrifuged (10 min at 4500 rpm). The fluid was tested using UV-Vis to determine the amount of the drug retained, while the carrier was characterized by other techniques and used in the drug release step. Five repetitions were made.

The material after MERC sorption was named mSiO2-PDA-Zn-MERC.

Sorption was also tested for the unmodified material, but it was not effective, and the results for this type of material are not presented.

### 2.5. MERC Release

mSiO2-PDA-MERC samples (15 mg) were placed in a vial with 1 mL of simulated body fluid (SBF) with a pH of 7.4 and a temperature of 36.6 °C. The amount of the drug released was measured after each hour using UV-Vis spectroscopy. Each time, the samples were centrifuged (10 min with 4500 rpm), and the SBF was replaced with a new portion to supply new ions. The addition of a new portion of SBF imitates blood flow, and this method was used due to the potential use of the material in intravenous administration. Three repetitions were made. The composition of the SBF is given in Table S1.

### 2.6. Characterization of the Carrier

The morphology of the samples was analyzed using a Scanning Electron Microscope Quanta FEG 250 (FEI) equipped with an EDS Octane SDD detector (EDAX). The structure of bulk samples and EDS mapping analyses were conducted in the Low Vacuum mode at the pressure of 70 Pa with an accelerating voltage of 10 kV, while the structure of individual mSiO2-PDA-Zn carriers was visualized after deposition from water dispersion at the silicon wafer and realized in the high vacuum mode, with an accelerating voltage of 30 kV. The size distribution and Zeta potential analyses were performed using a Zetasizer Nano ZS analyzer (Malvern Instruments Ltd.) operating at room temperature. Size distribution analyses were realized by a Dynamic Light Scattering (DLS) technique using non-invasive backscatter technology, with a 173° detection angle. All DLS and Zeta potential measurements were performed in a deionized water environment at a pH equal to 7. The tested material (1 mg/mL) was sonicated in an ultrasonic bath in distilled water for 3 min. Five repetitions were made for each material. Fourier-Transform Infrared Spectroscopy (FT-IR) analysis was performed using a Vertex70 spectrometer (Bruker Optics). The materials were tested using a single reflection diamond ATR crystal. The tests were carried out in the spectral range of 4000–800 cm^−1^, with a resolution of 4 cm^−1^ and 32 scans for signal accumulation. Elemental analysis was performed on the FLASH 2000 elemental analyzer. Thermogravimetric analysis was conducted using the TGA 4000 analyzer (Perkin Elmer), with a 20 mL/min nitrogen flow and 10 °C/min heating rate. The UV-Vis spectrophotometer UV-2600 (Shimadzu, Japan) was applied for the determination of the MERC concentration during the sorption and release process. Measurements were made in the range of 300–400 nm (λ max = 320 nm). Zinc ion release measurements were carried out on a mass spectrometer with induction-induced plasma ICP-MS NexION 300d (PerkinElmer). The liquid samples were subjected to quantitative analysis. For this purpose, a calibration curve was made, and the zinc concentration was determined on the basis of it. To examine the statistically significant differences, one-way variance analysis was carried out at the significance level of 0.05. All calculations were performed with the use of Statistica 13.1 software (TIBCO Software).

## 3. Results

### 3.1. Characterization of Synthesized Materials Using SEM Analysis

**Figure 2:**
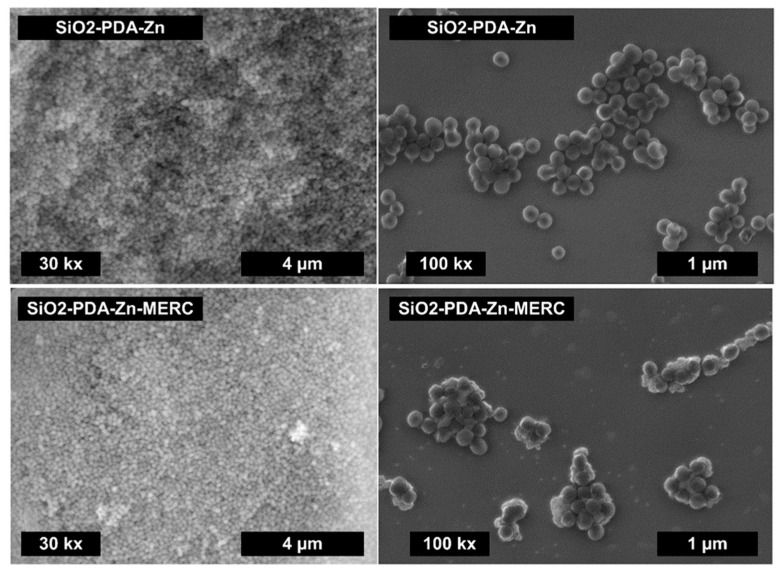
SEM images for the mSiO2-PDA-Zn carrier before and after the sorption of MERC.

To confirm the effectiveness of the synthesis of polydopamine-modified mesoporous silica, we carried out SEM imaging (Figure 2). The synthesized drug carrier has a typical spherical shape, which is consistent with the results obtained by Shen et al. [30]. Only spherical particles are visible in the photo, which means that polydopamine has not formed in other forms, except for the mesoporous silica structure. The images obtained by SEM at a lower magnification do not show significant differences in the morphology of the mSiO2-PDA-Zn carrier before and after drug sorption (Figure 2). As the agglomeration of the carrier means that it should not be used for intravenous delivery [45,46], we analyze the morphology of the water dispersion of mSiO2-PDA-Zn carriers. Higher-magnification SEM images, obtained from dispersion deposited on the silicon wafer, reveal that the spherical-shape particles do not form huge aggregates, which was confirmed by DLS (see below). Only the flat, interconnected particles are visible in both samples, which can be the result of sample drying. In some places, the surface of the carrier with the drug is not as smooth, which most likely indicates the partial precipitation of the drug on its surface. However, it is worth noting that we do not see any crystalline structures that are not bound to the carrier. The lack of carrier agglomeration and nonprecipitation of the drug is very important, as these phenomena could result in the inability to use this material in the delivery of MERC [47,48].

### 3.2. Determination of the Number and Distribution of Elements Using EDS Analysis

**Figure 3:**
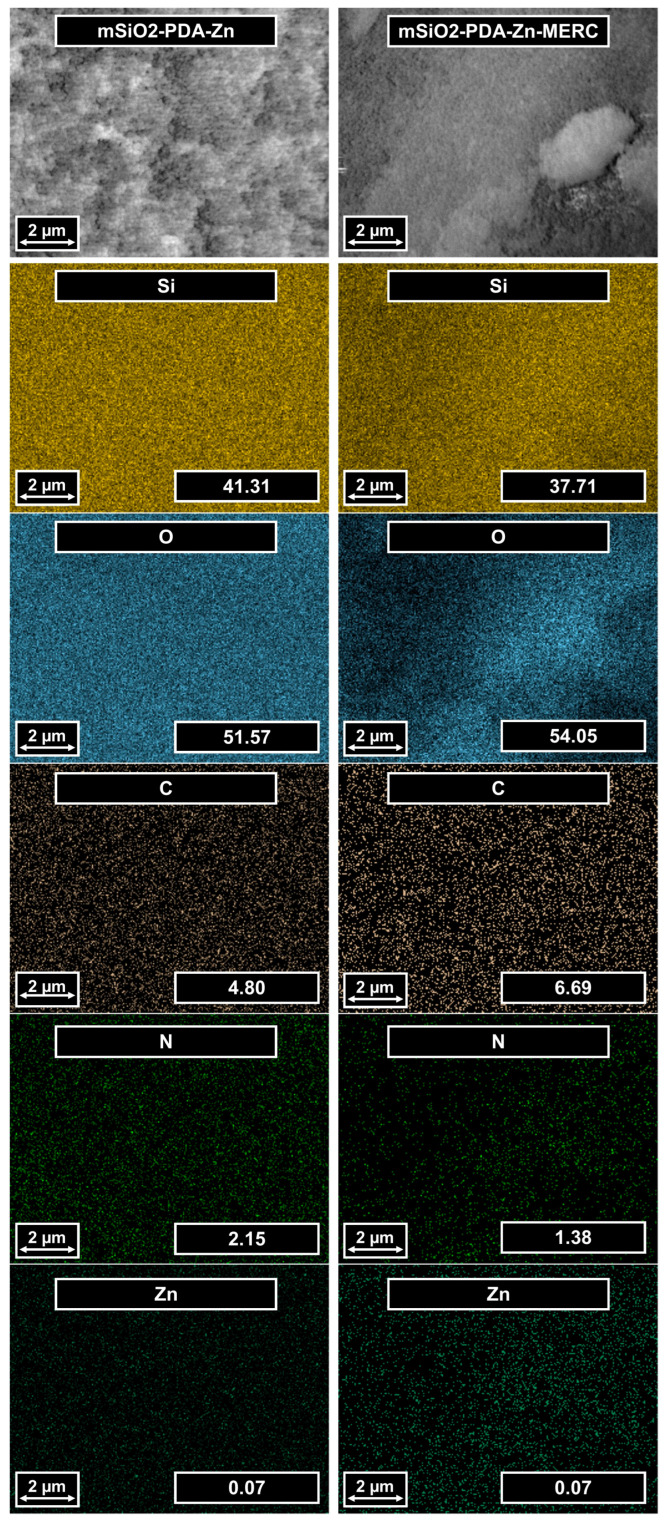
SEM images of the carrier before and after MERC sorption (first row). Elemental mapping of the same regions indicating the spatial distribution of silica, oxygen, carbon, nitrogen, and zinc. The values in the images represent the % by the weight of the elements.

The number and distribution of elements in the synthesized materials were determined using EDS (Figure 3). Both samples contain the same elements: silica, oxygen, carbon, nitrogen, and zinc. Oxygen and silicon come from mesoporous silica—the main component of this carrier, which is indicated by the number of these elements. Both materials contain carbon and nitrogen, which are the constituents of polydopamine. Small amounts of zinc were also noted. Such a small amount of zinc is consistent with the results described by Shen et al. [30]. Based on the X-ray photoelectron spectroscopy (XPS) results, they determined that 0.34% Zn is on the surface. The greater content compared to that in this work is due to the fact that the EDS technique determines the ion content deeper than the XPS technique [49,50]. Zinc ions are on the surface, so their amount in the presented work is smaller. It is worth noting that the presented maps do not show any agglomeration of any of the elements. This is important because it indirectly indicates that the prepared materials are homogeneous, which is very important for materials with potential drug release applications.

### 3.3. Determination of the Zeta Potential and Particle Size of Synthesized Materials

**Figure 4:**
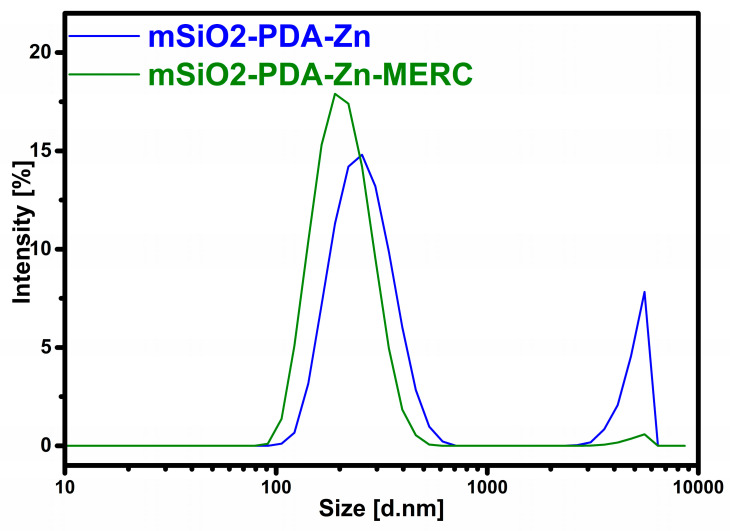
Particle size distribution by intensity.

The size of the prepared materials was also determined using DLS analysis, in addition to SEM analysis. The susceptibility of the materials to particles agglomeration was determined using the zeta potential analysis. The results of both analyses are presented in Table 1. The results obtained indicate, to some extent, the effectiveness of obtaining a polydopamine layer on the surface of the mesoporous silica because the silica synthesized by this procedure without the addition of polydopamine has a zeta potential close to −20 mV and a particle size of about 330 nm [30]. The values obtained in this work are closer to those of the materials with a polydopamine layer [30]. On the basis of the zeta potential results, it can also be seen that the mSiO2-PDA-Zn material tends to agglomerate slightly more than the mSiO2-PDA-Zn-MERC material. This indicates the effectiveness of coating the carrier with a drug layer. The drug layer does not have adhesive properties like the polydopamine layer. The zeta potential value for the material mSiO2-PDA-Zn-MERC is close to −30 mV, a value indicating that the material does not agglomerate at a neutral pH [51,52]. The results of the particle size distribution may seem illogical, as the samples after the sorption of the drug reduce in size. This is due to the fact that carrier particles without the drug are more likely to slight agglomeration, so the final averaged results are higher for them. Most importantly, the particle size of the mSiO2-PDA-Zn-MERC material is close to 200 nm, and therefore, the material can be used intravenously [47,48]. The differences in particle size can also be explained by the results shown in Figure 4. As you can see for both samples, we have two visible peaks, the first one around 200 nm and the second one around 5000 nm. The occurrence of the second peak affects the increase in the average particle diameter as well as the value of the polydispersity index. The value of the polydispersity index is lower for mSiO2-PDA-Zn-MERC (0.226) than for mSiO2-PDA-Zn (0.563) (Table 1). This indicates that the particle sizes for the mSiO2-PDA-Zn-MERC material are more similar to each other, which is consistent with the conclusions obtained from the analysis of Figure 4. From the SEM results, it could be seen that the carrier particles are generally of the same shape and size, so most likely, simply filtering or sonicating the carrier suspension prior to the formulation would solve the problem of the presence of large particles.

### 3.4. Analysis of Synthesized Materials Using the FT-IR Technique

**Figure 5:**
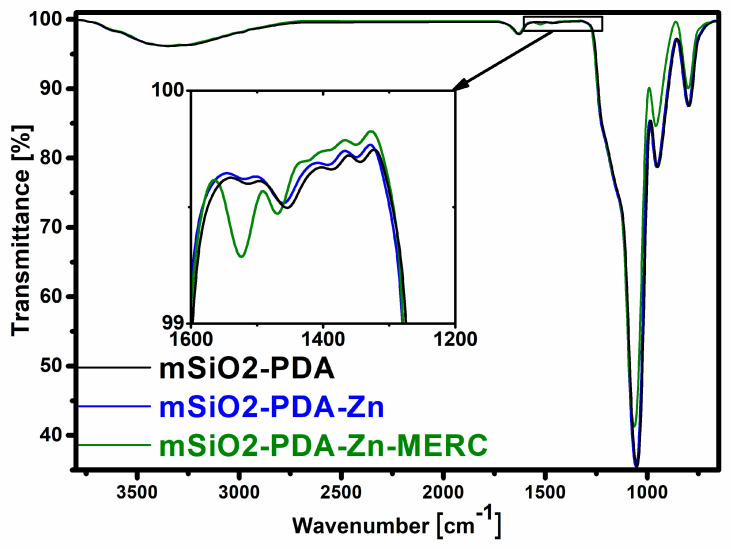
FTIR spectra of the carrier before and after sorption.

The efficiency of carrier synthesis and drug adsorption was also confirmed by FTIR analysis (Figure 5). In both materials, there is a wide band at the wavenumber of approximately 3300 cm^−1^ that is attributed to the stretching vibrations of Si-OH and water adsorbed on the carrier surface [53]. The band at the wavenumber of 1630 cm^−1^ is also attributed to the adsorbed water, while the band located at 950 cm^−1^ is attributed to the bending vibrations of Si-OH. Two characteristic peaks of the asymmetric stretching vibration of Si-O-Si appeared at 1056 cm^−1^ and 800 cm^−1^ [22]. The effectiveness of silica modification with polydopamine is confirmed by the bands at 1455 cm^−1^ (N-H stretching vibration) and 1390 cm^−1^ (phenolic C-O-H bending vibration) [31,54]. The small bands of PDA are also located at 1510 cm^−1^ (N-H shearing vibration) and at 1347 cm^−1^ (CH_2_ bending vibration) [22,55]. The spectra for the carrier before and after zinc sorption are similar. As seen in the presented spectra, new bands appear after drug sorption, confirming the drug’s effective sorption on the carrier surface. The band after drug sorption at the wavenumber of 1524 cm^−1^ can be attributed to the N-H bending vibration, while the band at 1470 cm^−1^ can be attributed to the N-H stretching vibration [56,57].

### 3.5. Elemental Analysis of Synthesized Materials

The amount of nitrogen, carbon, and hydrogen in the synthesized materials was determined by elemental analysis (Table 2). The amount of nitrogen in mSiO2-PDA-Zn could not be determined using this technique. This is most likely due to the fact that the content of this element is too low. In polydopamine, the nitrogen content is approximately seven times lower than that of carbon; therefore, it may be too small to be determined in this material [58]. Nitrogen was successfully determined for the material after drug sorption. The amount of nitrogen in the drug structure is greater than that in polydopamine. This is because there is more nitrogen in the drug than in polydopamine, and the carbon-to-nitrogen ratio is approximately one [59]. After the sorption of the drug, an increase in the amount of carbon was also observed, which may indicate, to some extent, that the polydopamine layer does not degrade during the sorption process. The hydrogen present in the materials comes from not only the carrier and the drug but also from the absorbed water. The increase in the amount of nitrogen and carbon after drug sorption is statistically significant.

### 3.6. Characterization of Materials Using Thermogravimetric Analysis

**Figure 6:**
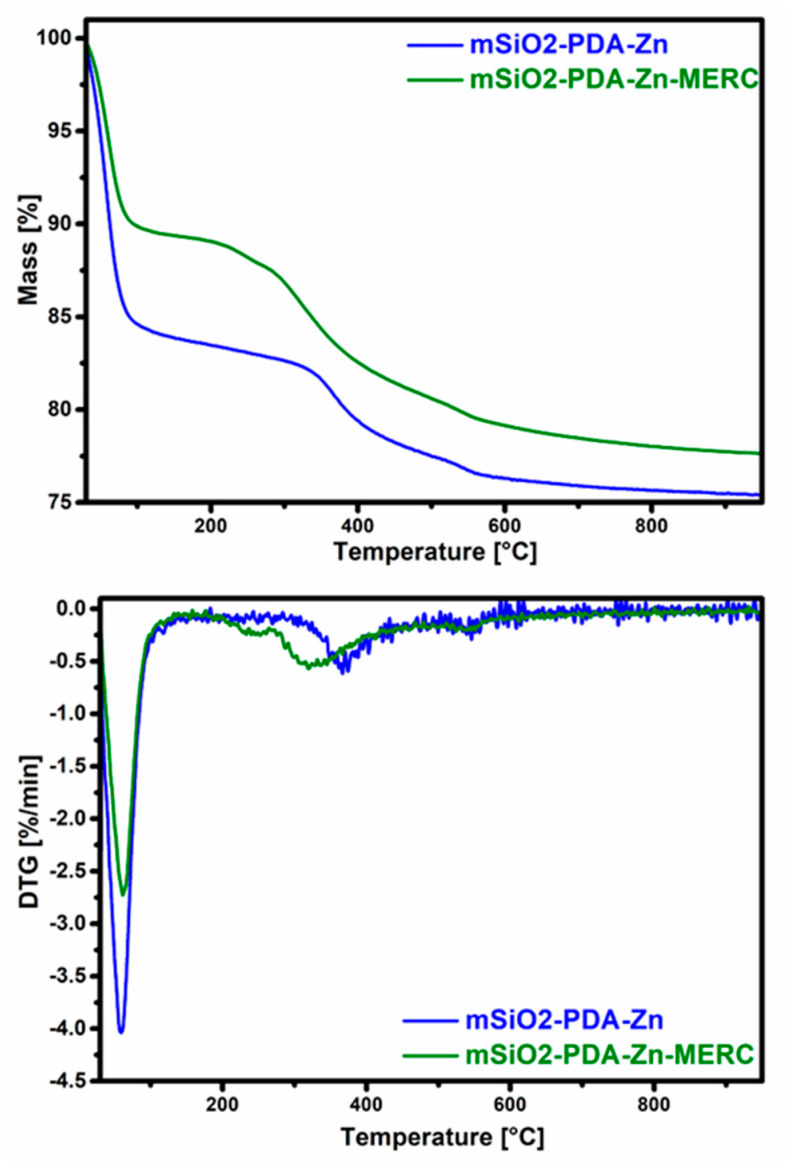
Thermogravimetric (**top**) and derivative thermogravimetric curves (DTG, (**bottom**)) of examined materials.

Both materials were also characterized using thermogravimetric analysis (Figure 6, Table 3). As can be seen from the diagram, mSiO2-PDA-Zn is less stable than mSiO2-PDA-Zn-MERC. This is surprising, since the material with the adsorbed drug should degrade to a greater extent than that without the adsorbed drug. As can be seen, mSiO2-PDA-Zn has a large weight loss in the range of 30–150 °C and the next after reaching 350 °C. In the case of mSiO2-PDA-Zn-MERC, there is another starting at about 250 °C.

To understand more precisely why the greatest mass loss occurs for the mSiO2-PDA-Zn material, derivative thermogravimetric curves should be analyzed (Figure 6). As can be seen in Figure 6, the weight loss for mSiO2-PDA-Zn is greater up to a temperature of 150 °C; therefore, it is only due to the higher water content. The higher water content also explains the higher hydrogen content found in the elemental analysis (Table 2). The results obtained also indicate the effectiveness of the formation of the polydopamine layer and drug sorption. A weight loss at about 360 °C for the material mSiO2-PDA-Zn indicates the presence of polydopamine [60]. For the material mSiO2-PDA-Zn-MERC, the weight loss starts earlier. The mass reduction step at 325 °C for mSiO2-PDA-Zn-MERC probably corresponds to the decomposition of MERC, and these results are similar to those described by Doriani et al. [61]. The low weight loss for mSiO2-PDA-Zn-MERC at about 240 °C indirectly indicates the presence of a complex between mercaptopurine and zinc, as extensively described in the publication of Sharfalddin et al. [21]. To better determine the mass loss of materials with the exclusion of water, the mass loss was calculated in two ranges: 30–150 °C and 150–800 °C (Table 3). In the first range, a greater loss is observed for mSiO2-PDA-Zn. In the second range, for mSiO2-PDA-Zn, the loss is 8.2%, and for mSiO2-PDA-Zn-MERC, the loss is 11.4%, which indicates the effectiveness of drug retention.

### 3.7. Drug Sorption

**Figure 7:**
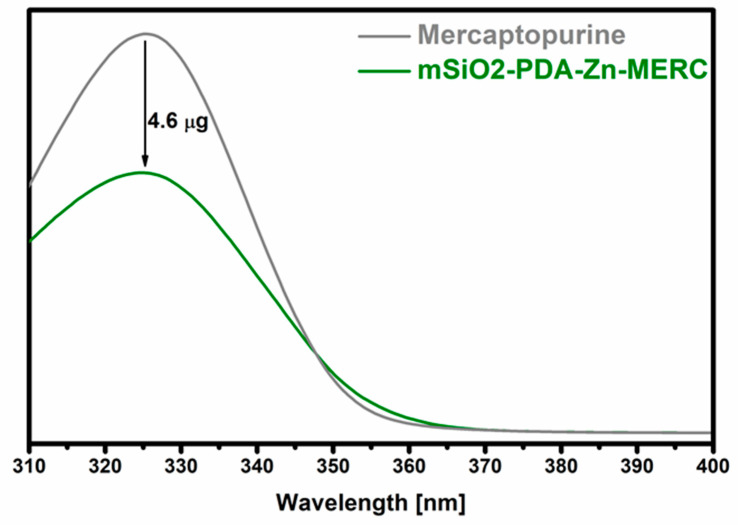
Sorption of mercaptopurine on the surface of the mSiO2-PDA-Zn carrier. mSiO2-PDA-Zn-MERC is the amount of the drug remaining after sorption. “Mercaptopurine” means the starting solution before the sorption of the drug on the carrier. The difference between the two values indicates the amount of the drug retained.

During the research, the ability of the synthesized carrier to retain MERC was checked. The maximum amount of the drug that could be adsorbed was 4.6 µg ± 0.5 (Figure 7). The amount of the adsorbed drug is statistically significant.

### 3.8. Release of the Drug and Zinc Ions from the Carrier

**Figure 8:**
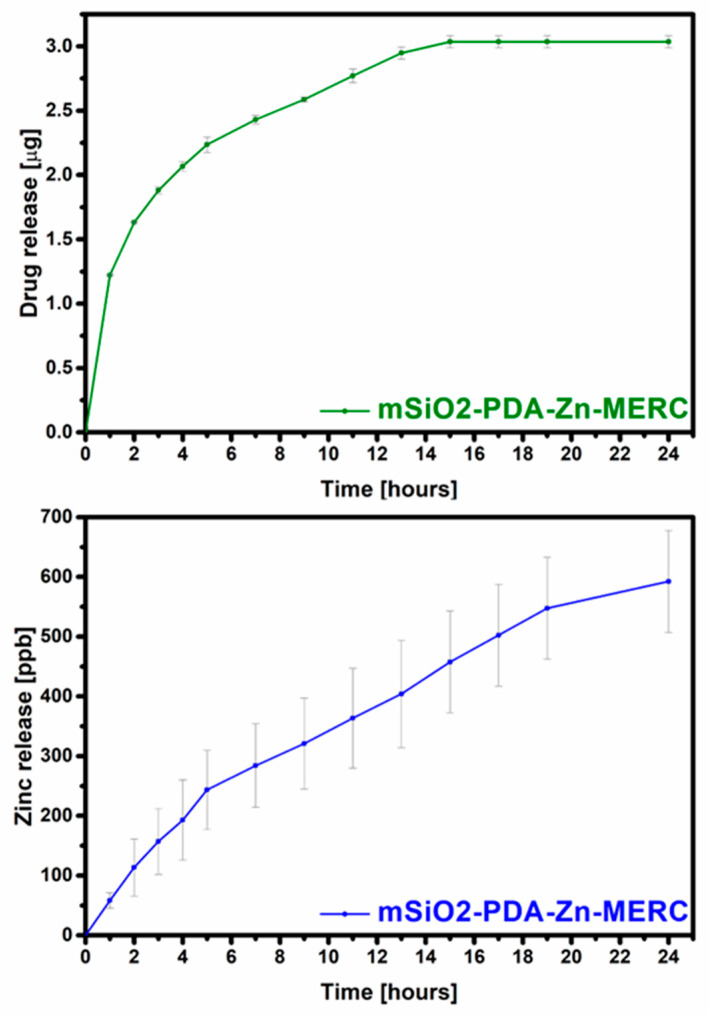
Total release of MERC (**top**) and zinc (**bottom**) from the carrier under the influence of SBF.

More important information than the amount of the drug adsorbed is how much of the drug will be released and at what time. In the case of MERC, it is important that the carrier provides sustained drug release that may increase bioavailability. As can be seen in Figure 8, the carrier presented in this work released the drug over 15 h. The amount of the drug released after 15 h is approximately 66% of the total amount retained in the carrier. After this hour, no drug was released, or it was released in too small of an amount to be determined by the technique used. The variation between the results can be considered as small, as it is, on average, below 1.5%, which is important because the drug from any amount of the carrier should be released at a comparable dose to counteract possible inflammation and toxic reactions. The highest amount of the drug was released in the first hour (~25%). Based on the release profile obtained, it was confirmed that this carrier affects the sustained release of the drug and is not characterized by the “burst release” found in many types of carriers [62].

During the research, the amount of zinc released from the carrier was also determined (Figure 8). As can be seen from the graph, zinc is gradually released. This is very important, as the uncontrolled rapid release of zinc can disrupt zinc homeostasis, leading to protein dysfunction [63,64]. From these results, it can be seen that both the drug and ions are not released in high doses. The number of ions is so low that it will not cause side effects during intravenous delivery and, therefore, can only have a positive effect on the patient’s health [65,66,67]. As can be seen from the zinc ion release curve, it will continue to be released after 24 h, but its doses are not toxic to the human body [65].

## 4. Conclusions

In the presented work, it was possible to obtain the drug carrier, which was polydopamine-modified mesoporous silica with adsorbed zinc ions. The carrier is a biocompatible material, which has already been described by many research teams. The carrier was used for the first time as a material with potential use in the sustained release of mercaptopurine. The results presented confirm the effective preparation of the spherical carrier particles. No impurities were observed in the obtained material; the carrier did not agglomerate before and after sorption. Due to its size (100–150 nm), the carrier can be used to deliver drugs intravenously. The release of the drug was for 15 h, so the drug will be released into the bloodstream. Zinc ions were also released from the carrier in small doses. The results obtained are promising and have great application potential. The release of both mercaptopurine and zinc ions may provide a dual therapeutic effect against ALL. The material obtained in the next stages of research should be tested in vitro and in vivo to determine its effect on cells.

## Figures and Tables

**Table 1:** Zeta potential, particle diameter (by number), and polydispersity index (PDI) of the obtained materials. Particle diameters were determined by DLS.

	Zeta Potential (mV)	Particle Diameter (nm)	PDI
mSiO2-PDA-Zn	−27.7	203.0	0.563
mSiO2-PDA-Zn-MERC	−29.5	163.2	0.226

**Table 2:** Elemental analysis of the carrier before and after drug sorption.

	N	C	H
mSiO2-PDA-Zn	0	3.08 ± 0.04	1.74 ± 0.25
mSiO2-PDA-Zn-MERC	0.81 ± 0.01	4.53 ± 0.14	1.06 ± 0.11

**Table 3:** Weight loss in the temperature ranges for mSiO2-PDA-Zn and mSiO2-PDA-Zn-MERC.

	30–150 °C	150–800 °C
mSiO2-PDA-Zn	16.1%	10.6%
mSiO2-PDA-Zn-MERC	8.2%	11.4%

## Data Availability

All data generated or analyzed during this study are included in this published article.

## Supplementary Materials

The following supporting information can be downloaded at: https://www.mdpi.com/article/10.3390/ma16124358/s1, Table S1: Composition of the SBF used in this work (1000 mL of the SBF).
