# Supplementary material for: Mesoporous Silica Modified with Polydopamine and Zinc Ions as a Potential Carrier in the Controlled Release of Mercaptopurine
Source: Materials (Basel). 2023 Jun 13;16(12):4358. doi: 10.3390/ma16124358 (PMC10301584; doi:10.3390/ma16124358)
Supplement: Supplementary file 1 [file materials-16-04358-s001.zip › materials-2422148-supplementary.pdf]

Supplementary Material

Mesoporous Silica Modified with Polydopamine and Zinc Ions as a Potential Carrier in the Controlled Release of Mercaptopurine

Mariusz Sandomierski [1,*], Martyna Chojnacka [1], Maria Długosz [1], Monika Pokora [2], Joanna Zwolińska [2], Łukasz Majchrzycki [2] and Adam Voelkel [1]

[1] Institute of Chemical Technology and Engineering, Poznan University of Technology, ul. Berdychowo 4, 60-965 Poznań, Poland
[2] Center for Advanced Technologies, Adam Mickiewicz University, Poznań, ul. Uniwersytetu Poznańskiego 10, 61-614 Poznań, Poland
* Correspondence: mariusz.sandomierski@put.poznan.pl

Table S1. Composition of the SBF used in this work (1000 ml of the SBF).

Order	Reagent	Amount
1	NaCl	8.035 g
2	$NaHCO_3$	0.355 g
3	KCl	0.225 g
4	$K_2HPO_4·3H_2O$	0.231 g
5	Na_2SO_4	0.072 g
6	TRIS	0.6112 g
7	HCl	0-5 ml
